# RETRACTION: Ellagic Acid Reduces Adipogenesis through Inhibition of Differentiation‐Prevention of the Induction of Rb Phosphorylation in 3T3‐L1 Adipocytes

**DOI:** 10.1155/ecam/9784084

**Published:** 2026-11-03

**Authors:** Evidence-Based Complementary and Alternative Medicine

RETRACTION: L. Wang, L. Li, X. Ran, et al., “Ellagic Acid Reduces Adipogenesis through Inhibition of Differentiation‐Prevention of the Induction of Rb Phosphorylation in 3T3‐L1 Adipocytes,” *Evidence-Based Complementary and Alternative Medicine*, 2013287534, https://doi.org/10.1155/2013/287534.

The above article, published online on 04 November 2013 in Wiley Online Library (https://wileyonlinelibrary.com), has been retracted by John Wiley & Sons Ltd. The retraction has been agreed following figure concerns raised by the University of Wyoming and on PubPeer [1]. Specifically:

• There are unexpectedly repeated areas between the Control and DMSO panels on the top row in Figure 2a
• There are unexpectedly repeated areas between the Control and DMSO panels on the second row in Figure 2a
• There are unexpectedly repeated areas between the Control panel on the second row in Figure 2a and the EA 20 µM + MDI d6‐8 microscopy image shown in Figure 3a
• There are unexpectedly repeated areas between the DMSO panel on the second row in Figure 2a and the EA 20 µM + MDI d6‐8 microscopy image shown in Figure 3a
• The Control panel in the bottom row of Figure 2a is identical to the EA 20 µM + MDI d4‐8 plate shown in Figure 3a
• There are unexpectedly repeated areas between the EA 15‐µM panel on the bottom row in Figure 2a and the EA 20 µM + MDI d2‐8 plate image shown in Figure 3a (after rotation)
• The EA 20‐µM panel in the bottom row of Figure 2a is identical to the EA 20 µM + MDI d0‐8 plate shown in Figure 3a
• In Figure 3a, the Control and EA 20 µM + MDI d4‐8 microscopy images appear identical (d4‐8 slightly zoomed in)
• The EA 15 µM panel in the second row of Figure 2a is identical to the Figure 3g panel in [2].
• The DMSO panel in the second row of Figure 2a is identical to the Figure 3f panel in [2].
• There are unexpectedly repeated areas between the EA 20 µM + MDI d2‐8 microscopy image in Figure 3a and Figure 3i in [2].
• The ß‐actin bands shown in Figure 3b appear identical to the first five ß‐Actin bands shown in Figure 6c
• There are unexpectedly repeated areas between the EA 20 µM + Hoechst 33342 panels at 16 hours and 20 hours in Figure 4e
• The ß‐actin bands shown in Figure 5a and 5c are identical
